# The Influence of Gut Microbiota on Oxidative Stress and the Immune System

**DOI:** 10.3390/biomedicines11051388

**Published:** 2023-05-08

**Authors:** Claudia Kunst, Stephan Schmid, Marlen Michalski, Deniz Tümen, Jonas Buttenschön, Martina Müller, Karsten Gülow

**Affiliations:** Department of Internal Medicine I, Gastroenterology, Hepatology, Endocrinology, Rheumatology and Infectious Diseases, University Hospital Regensburg, 93053 Regensburg, Bavaria, Germany; claudia.kunst@ukr.de (C.K.); stephan.schmid@ukr.de (S.S.); marlen.michalski@gmail.com (M.M.); deniz.tuemen@ukr.de (D.T.); jonas.buttenschoen@ukr.de (J.B.); martina.mueller-schilling@ukr.de (M.M.)

**Keywords:** intestine, microbiome, reactive oxygen species (ROS), REDOX, inflammation, dysbiosis

## Abstract

The human gastrointestinal tract is home to a complex microbial community that plays an important role in the general well-being of the entire organism. The gut microbiota generates a variety of metabolites and thereby regulates many biological processes, such as the regulation of the immune system. In the gut, bacteria are in direct contact with the host. The major challenge here is to prevent unwanted inflammatory reactions on one hand and on the other hand to ensure that the immune system can be activated when pathogens invade. Here the REDOX equilibrium is of utmost importance. This REDOX equilibrium is controlled by the microbiota either directly or indirectly via bacterial-derived metabolites. A balanced microbiome sorts for a stable REDOX balance, whereas dysbiosis destabilizes this equilibrium. An imbalanced REDOX status directly affects the immune system by disrupting intracellular signaling and promoting inflammatory responses. Here we (i) focus on the most common reactive oxygen species (ROS) and (ii) define the transition from a balanced REDOX state to oxidative stress. Further, we (iii) describe the role of ROS in regulating the immune system and inflammatory responses. Thereafter, we (iv) examine the influence of microbiota on REDOX homeostasis and how shifts in pro- and anti-oxidative cellular conditions can suppress or promote immune responses or inflammation.

## 1. Introduction

Reactive oxygen species (ROS) are generated in living cells. Initially, it was discovered that ROS are formed as by-products of enzymatic reactions. However, a few years later, it became evident that ROS are also generated in a controlled manner by eukaryotic cells. Thus, it was obvious that ROS not only are harmful by-products but also exert physiological functions as intracellular and intercellular messengers. Various proteins can be modified by ROS, for example, p53, Jun, Fos, and the NF-κB subunits p50 and p65. The oxidation of these proteins leads either to the stimulation (p50) or inhibition (p53, Jun, Fos, and p65) of these proteins; thus, ROS play an important role in intracellular signaling [1,2]. Long-lived ROS that can cross membranes also affect neighboring cells. Thus, ROS also play a role in the communication between cells [1,3]. To enable these signaling functions and prevent oxidative damage, the balance between pro-oxidative and antioxidative molecules must be strictly controlled. If this REDOX equilibrium is disturbed, oxidative stress and cell and tissue damage will occur [4].

Oxidative signals are particularly important for the activation of our immune system. Here ROS are generated by a metabolic switch from cellular respiration to glycolysis. ROS production is essential for the regulation of an appropriate immune response [5,6,7]. In addition, ROS can also be used by antigen-presenting cells (e.g., monocytes and B cells) and neutrophils as a defense mechanism against pathogens [6].

The gut microbiome is unique to each individual. There appears to be a balance in composition and diversity that is beneficial for the host and suppresses inflammation. The intestine is the only place where continuous activation of the immune system through direct contact with microbiota occurs. Under physiological conditions, there is a balance of pro- and anti-inflammatory mechanisms. This balance is maintained by microbiota influencing the REDOX system. Commensal bacteria often exhibit anti-oxidative properties and suppress inflammatory reactions. Pathogenic microbiota induce inflammation and shift the REDOX balance toward a pro-oxidative status [8]. Therefore, the interaction between the intestinal microbiota and the host’s cells, especially the immune cells, is crucial in maintaining the REDOX equilibrium and suppressing unwanted inflammation.

## 2. The Cellular REDOX Equilibrium

In a physiological state, cells display a balanced REDOX equilibrium. This depends on one hand on the production of reactive molecules and on the other hand on the oxidative defense. Under physiological conditions, the REDOX balance allows oxidative signaling while inhibiting oxidative damage. Under pathophysiological conditions, this balance can shift so that either cellular signaling is impaired and/or oxidative damage is promoted. This is referred to as oxidative stress.

### 2.1. Reactive Oxygen (ROS)

To date, the best-characterized ROS include superoxide anions (O_2_·^−^), hydroxyl radicals (·OH), and hydrogen peroxide (H_2_O_2_) [4,5]. Cellular ROS production starts with the transfer of an electron to oxygen. This leads to the formation of O_2_·^−^. Due to their energetically unstable state, these molecules are highly reactive and have only a short half-life of about 1 µs. Furthermore, they cannot freely cross cellular membranes due to their charge. As a result, O_2_·^−^ has a locally limited effect and is responsible for oxidative damage rather than acting as a signaling molecule [1,7].

In an aqueous environment, O_2_·^−^ rapidly converts to H_2_O_2_. Intracellularly, this process is accelerated by superoxide dismutases (SODs) [9,10,11]. Although H_2_O_2_ is not a radical, it is classified as an ROS. H_2_O_2_ displays a longer half-life of about 1 ms compared with O_2_·^−^. In addition, H_2_O_2_ can diffuse freely through membranes similar to water, and it targets mainly free thiols. This oxidation is generally reversible, which means that H_2_O_2_ fulfills the requirements for a secondary messenger [1,6,7,12]. To prevent excessively high H_2_O_2_ concentrations and thus oxidative damage, the amount of H_2_O_2_ is strictly controlled by enzymes such as catalase or in addition by thiol scavengers [13].

The accumulation of intracellular H_2_O_2_ increases the risk of a Fenton reaction [14], which can cause major cell and tissue damage. In this reaction, H_2_O_2_ interacts with free iron (Fe^2+^) to produce highly reactive ·OH [10,15,16]. The majority of iron in the cell is present in the bound form as Fe^3+^, which is unreactive. However, there is always a small amount of free Fe^2+^ in the cell, the so-called labile iron pool. In the case of excessive amounts of H_2_O_2_, Fe^2+^ and H_2_O_2_ can react with each other. The resulting ·OH is extremely reactive [1]. The oxidation of cellular macromolecules, especially lipids, by ·OH leads to uncontrollable chain reactions and massive cell and tissue damage, followed by inflammatory reactions [10,16,17].

In addition to ROS, nitric oxide (NO) can be generated in mammalian cells by oxidation of one of the terminal guanidino nitrogen atoms of L-arginine. This reaction is catalyzed by the enzyme NO synthase (NOS) [18,19]. Nitric oxide itself is less reactive and generally not harmful. However, if NO molecules accumulate, they rapidly react with O_2_·^−^ to form highly detrimental peroxynitrite (ONOO^−^) [3,7]. Peroxynitrite reacts with multiple substrate derivatives and can induce cellular damage, whereas NO itself functions as a second messenger [20]. In this review article, we focus on ROS and its physiological and pathophysiological functions.

### 2.2. Sources of ROS Generation

**Figure 1:**
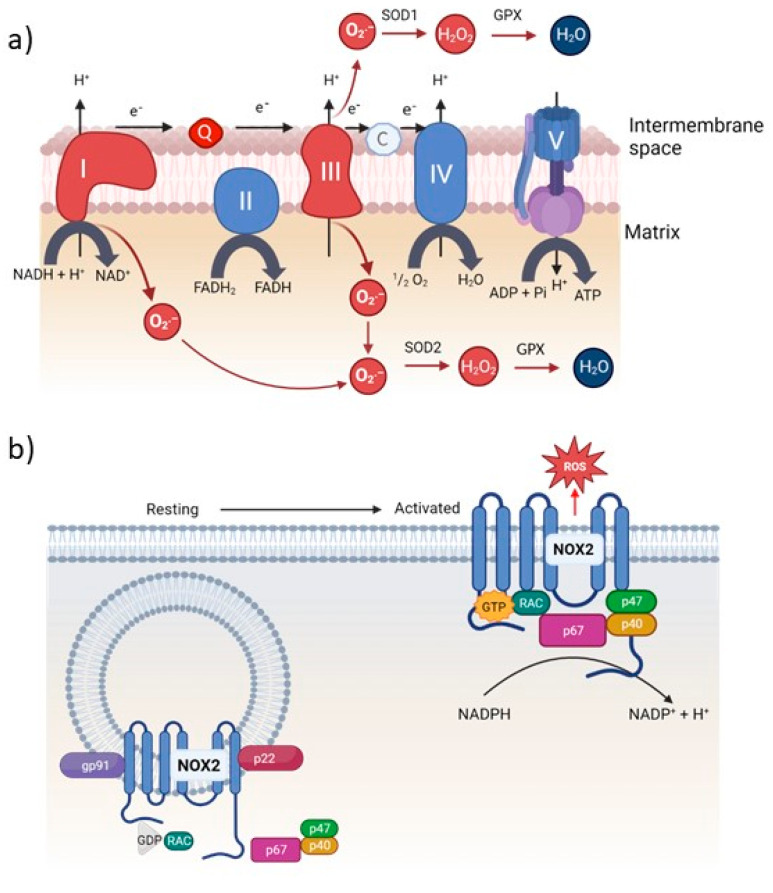
(**a**) Schematic illustration of the mitochondrial respiratory chain. Electrons are fed into the respiratory chain via NADH in complex I or via FADH_2_ in complex II. The electrons are passed through the respiratory chain and transferred to O_2_, resulting in the formation of water. However, some of the electrons can leave the respiratory chain, leading to a univalent reduction of oxygen and O_2_·^−^ formation. O_2_·^−^ is converted into H_2_O_2_ by SODs. (**b**) Scheme of phagocytic NADPHoxidase. The phagocytic NADPHoxidases are multicomponent complexes that catalyze a one-electron reduction of oxygen by NADPH. The resulting O_2_·^−^ is generated to the outside of the cell or to the inside of phagosomes. Q: ubiquinol; C: cytochrome C; gp: glycoprotein; RAC: subfamily of the Rho family of GTPases. The figure was created with BioRender.com (accessed on 1 May 2023).

Among the many different cellular sources of ROS (Table 1), mitochondria and NADPHoxidases are the most important ones. In mitochondria, the electron transport chain (ETC) is responsible for the release of ROS (Figure 1a). The main site of univalent reduction of oxygen and thus the production of O_2_·^−^ is ubisemiquinone, a component of the ETC localized in the mitochondrial matrix [3,21,22,23]. In addition to this unintentional production of ROS via the ETC, ROS can also be generated in a controlled manner via complex I and complex III of the ETC. Thus, the mitochondrion can act as an oxidative signaling platform in many physiological settings, e.g., in the regulation of a T-cell immune response [7,24,25,26,27,28].

NADPHoxidases are multicomponent complexes that catalyze a one-electron reduction of oxygen by NADPH. Among the various NADPHoxidases within a cell, it is certainly the phagocytic NADPHoxidase, also called NADPHoxidase 2, that is most relevant to inducible ROS generation (Figure 1b) [29]. This plasma membrane-associated enzyme complex is best studied in phagocytes. However, it is also found in other cells, such as neutrophils, B lymphocytes, and dendritic cells. During the catalytic reaction, NADPHoxidase transports electrons across the plasma membrane to extracellular oxygen to form extracellular O_2_·^−^. These O_2_·^−^ are rapidly converted to H_2_O_2_, which can freely diffuse across the plasma membrane and thus translocate back into the cell [1,30]. NADPHoxidase 1 is another crucial ROS-producing enzyme. It is expressed in intestinal epithelial cells and plays an important role in cell migration, differentiation, and wound healing, and it can be induced by gut microbiota [29,31]. Like NADPHoxidase 2, NADPHoxidase 1 is a multicomponent membrane complex producing O_2_·^−^, which is immediately converted into H_2_O_2_. However, aberrant NADPHoxidase 1 activation or expression is involved in a growing number of diseases, including cancer [29,32].

**Table 1:** Cellular ROS sources influenced by gut microbiota.

Cellular Compartment	ROS Source	ROS	Mechanism of Action	ROS-Related Disease	Role of Gut Microbiota
Mitochondria	Complex I respiratory chain	O_2_·^−^	Cellular signaling, immune cell activation, energy metabolism	Inflammatory bowel disease (IBD) and colorectal cancer (CRC)	Induced by bacterial antigens, regulated by bacterial metabolites [7,33]
Mitochondria	Complex III respiratory chain	O_2_·^−^	Cellular signaling, immune cell activation, energy metabolism	IBD, CRC	Induced by bacterial antigens, regulated by bacterial metabolites [7,33]
Plasma membrane, vesicular membranes (endoplasmatic reticulum, endosome and lysosome)	NADPHoxidases	O_2_·^−^	Cellular signaling, host defense, immune cell activation, inflammation, oxidative burst	Inflammation, CRC, ileitis, and IBD	Induced by bacterial antigens [34,35,36]
Lysosome	Myeloperoxidase	H_2_O_2_	Neutrophile activation pathogen defense	IBD	Induced by bacterial antigens [37,38,39,40]
Peroxisomes	Flavoproteins include acyl-CoA oxidases, urate oxidase, D-amino acid oxidase, D-aspartate oxidase, L-pipecolic acid oxidase, L-α-hydroxyacid oxidase, and polyamine oxidase	H_2_O_2_	Combat/oxidize bacterial pathogens	IBD and CRC	Induced by bacterial antigens and metabolites [37,41,42,43]
Peroxisomes	Xanthine oxidase	O_2_·^−^/H_2_O_2_	Combat/oxidize bacterial pathogens, inflammation	Inflammation, type II diabetes	Induced by bacterial antigens and metabolites, purine degradation [37,41,42,43,44,45]
Cytosol	Xanthine oxidase	O_2_·^−^/H_2_O_2_	Inflammation	Inflammation, type II diabetes, gout	Purine degradation [37,44,45]

### 2.3. Oxidative Stress

Under physiologic conditions, free radicals and non-radical reactive compounds are present in tissues in low amounts. Their levels depend on the ratio of their production and degradation by anti-oxidants and anti-oxidative enzymes. This creates a REDOX equilibrium that allows oxidative signaling and prevents oxidation-induced damage. A shift of this REDOX equilibrium toward enhanced ROS production can result in unwanted and often non-reversible oxidation of macromolecules such as lipids, proteins, and DNA referred to as oxidative stress. However, it should be emphasized that the transition from REDOX balance to oxidative stress is different for each tissue and even from cell to cell, as it depends on the cellular background [3,4,37,46].

Antioxidants are classified as non-enzymatic or enzymatic. The most important non-enzymatic antioxidants include the tri-peptide glutathione and the proteins thioredoxin 1 and thioredoxin 2 [3,13]. Glutathione is a powerful antioxidant that can scavenge radical and non-radical reactive compounds such as H_2_O_2_, nitrites, nitrates, and benzoates. Thioredoxins display cytoprotective effects in various cellular responses by removing H_2_O_2_ and thus regulating the activity of redox-sensitive transcription factors, which often control the antioxidant defense system [2,13,47].

The major components of the enzymatic oxidative defense include catalase (CAT), superoxide dismutases (SOD1/2/3), glutathione peroxidase (GPX), and glutathione reductase (GSR) [13,48]. In particular, catalase and SODs form the main enzymatic defense against oxidative stress. SODs catalyze the conversion of O_2_·^−^ to H_2_O_2_, whereas CAT converts H_2_O_2_ to water [13,49]. GPX is part of the glutathione–REDOX system and converts glutathione to its oxidized form, thereby reducing H_2_O_2_ to water, and lipid hydroperoxides to their corresponding stable alcohols [13]. The GPX reaction is coupled to GSR, which reduces and thereby recycles oxidized glutathione [13].

## 3. REDOX Regulation of the Immune System

The state of the REDOX equilibrium is crucial for the immune system. Oxidative signals determine whether an immune response is switched on or off. To generate these important oxidative signals, most immune cells undergo a metabolic shift from mitochondrial respiration to glycolysis. This shift enables the cells to initiate oxidative signals. In addition, ROS are also used as a defense mechanism against pathogens. Here much higher concentrations of ROS are released by the cells to oxidize and destroy pathogens.

### 3.1. Activation-Induced Oxidative Signaling in Immune Cells

Oxidative signals in immune cells are very important. They not only regulate the expression of cytokines/interleukins (IL) but also control the differentiation into different cellular subsets. Here we describe the mechanisms of ROS generation in the most relevant immune cells.

#### 3.1.1. T-Cell Activation-Induced Oxidative Signaling

After T-cell receptor (TCR) stimulation, two signals are induced: (i) a calcium influx into the cytosol and (ii) initiation of an oxidative signal. The calcium influx leads to the activation of calcium-dependent transcription factors such as NF-AT or the activation of the neuronal nitric oxide synthase (nNOS) and the epithelial nitric oxide synthase (eNOS) [26]. However, the exact function of nNOS and iNOS in T-cell activation is still unclear [50,51,52]. The oxidative signal is initiated by a switch in the metabolism from mitochondrial respiration toward glycolysis comparable to the Warburg effect in cancer cells [24]. This results in a controlled release of O_2_·^−^ from mitochondria [24,26,28,53,54]. O_2_·^−^ are then converted to H_2_O_2_ by SODs [55], which then activate the REDOX-sensitive transcription factors NF-κB and AP-1. The oxidative signaling pathway can then be further significantly enhanced by an additional release of ROS by NADPHoxidase 2 [26,36]. NF-kappaB and AP-1 in concert with the calcium-dependent NF-AT constitute the minimal requirement for the induction of the expression of various cytokines or the CD95 death ligand (CD95L) and can thus control the induction and the termination of a T-cell immune response [7,24,25,26,56].

However, ROS play a crucial role not only in the activation of T cells but also in the CD4 T helper cell distribution. Thus, the activity, as well as the release of ROS from complex I, is a crucial factor for the differentiation of CD4^+^ cells to T_h_17 or T regulatory cells (T_reg_). It is not the function of the T-cell subsets that are affected but rather the differentiation of the different T-cell types [57,58]. Therefore, it can be stated that ROS and RNS make an important contribution to T-cell differentiation and activation.

#### 3.1.2. B-Cell Activation-Induced Oxidative Signaling

Unlike T cells, where an oxidative signal is first generated by the mitochondria, which is then amplified and prolonged by additional ROS production through the phagocytic oxidase 2, ROS are first produced by phagocytic NADPHoxidase after B-cell stimulation, and then the oxidative signal is prolonged by mitochondrial ROS generation [26,59]. In the absence of the second mitochondria-produced ROS signal, B-cell activation is inefficient, and cell proliferation is reduced [59,60]. Thus, cell mitochondrial ROS signaling participates in the activation processes of both B and T lymphocytes [61].

#### 3.1.3. Macrophages and Oxidative Signaling

Macrophages can be activated by commensal LPS, cytokines, pathogen-associated molecular patterns (PAMPs), damage-associated molecular patterns (DAMPs), and ROS. These warning signals induce transcription factors such as NF-κB, as well as PI3K and mTOR. Downstream pathways lead to the maintenance of macrophage activation and metabolic reprogramming. This shift from aerobic mitochondrial energy production to anaerobic glycolysis is essential in M1 macrophages for increased phagocytosis, increased production of inflammatory cytokines, and an even higher increase in ROS production [62,63,64,65,66,67].

In an environment with elevated levels of interleukin (IL)-4 and IL-13, M1 macrophages change their phenotype, become M2 macrophages, and develop anti-inflammatory and wound-healing properties [68,69]. During the shift from M1 to M2, the metabolism of macrophages switches back to mitochondrial energy production, and these cells become independent of oxidative signals [68,69]. In summary, inflammatory M1 macrophages are induced by ROS and produce ROS themselves, whereas anti-inflammatory M2 macrophages act independently of ROS. Of note, permanently elevated ROS levels and oxidative stress correlate with the induction of senescence [70,71,72] and may trigger cell death in macrophages [73,74].

#### 3.1.4. Dendritic Cells and Oxidative Signaling

Dendritic cells are typical antigen-presenting cells. Under physiological conditions, tissue-resident dendritic cells migrate to draining lymph nodes and present self-antigens, inducing tolerance [75]. However, after pathogen invasion, dendritic cells are activated via toll-like receptors (TLR), migrate to the lymph nodes, and undergo diverse changes in function and phenotype. Resting dendritic cells derive their energy from mitochondrial respiration. Upon activation by TLR stimulation, dendritic cells switch to glycolysis [76,77,78]. The intermediates produced by glycolysis are then shunted into the pentose phosphate pathway, which promotes the production of NO. NO can react with O_2_·^−^ to form highly reactive ONOO^−^, which in turn blocks the ETC and leads to an increase in ROS generation [79,80,81]. ROS production then has a decisive impact on antigen presentation and determines the activation of CD8^+^ and CD4^+^ T cells [82,83,84].

### 3.2. ROS, the Oxidative Burst, and the Inflammasome

Inflammation is primarily a host-induced defense against pathogens. The production of ROS is essential for the progression of the inflammatory reaction. Primarily, ROS are produced by cells of the immune system, mainly by antigen-presenting cells and polymorphonuclear neutrophils.

NADPHoxidases are certainly the main source of pathogen-induced ROS production. Currently, seven isoforms of NADPHoxidases are known (NADPHoxidase 1–5 and DUOX 1/2). NADPHoxidase 2 has the most important role in immune cells. Activation of ROS production induced by this NADPHoxidase 2 results in the release of high concentrations of O_2_·^−^. For example, neutrophils can produce ~10 nmol/min O_2_·^−^ per million neutrophils during the oxidative burst to oxidize and kill pathogens [85,86]. NADPHoxidase 2 deficiency leads to severe diseases such as chronic granulomatous disease [87]. In epithelial cells, NADPHoxidase 1 is mainly responsible for ROS production. The amounts of ROS produced by epithelial cells are much lower compared with an oxidative burst. Epithelial cell-derived ROS are not to oxidize and destroy bacteria directly; they rather work as messenger molecules controlling proliferation and cellular inflammatory responses [88,89].

**Figure 2:**
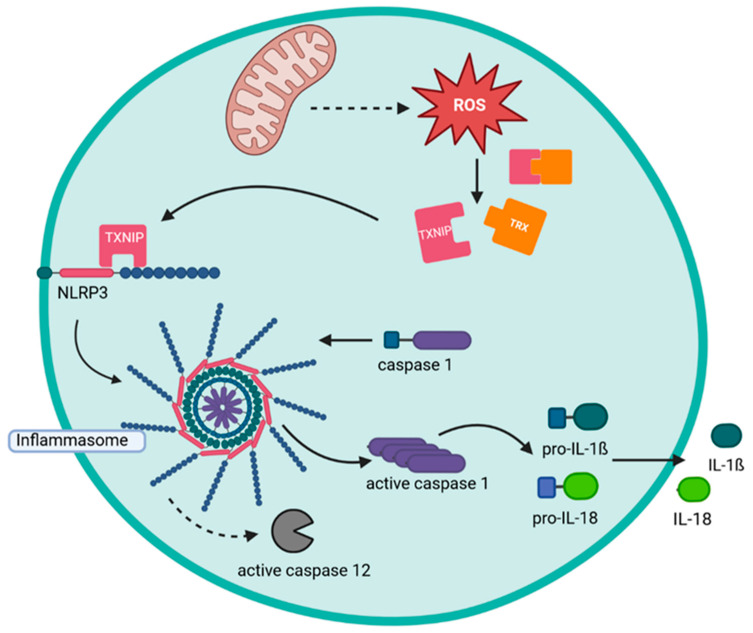
Formation of the NALP3 inflammasome. Mitochondria-derived ROS induce the release of TXNIP (VDUP-1) from the Thioredoxin/TXNIP complex. TXNIP (VDUP-1) binds to NALP3 and leads to the formation of the inflammasome. The NALP3 inflammasome induces activation of caspase 1 and caspase 12. These proinflammatory caspases in turn cleave and activate proinflammatory cytokines IL1β and IL18. NALP: NLR family pyrin domain containing; TRX: thioredoxin; TXNIP: thioredoxin interacting protein. The figure was created with BioRender.com (accessed on 1 May 2023).

Mitochondria-derived ROS also play an important role as messenger molecules in inflammatory responses. Thus, LPS can induce the production of mitochondrial ROS via TLR signaling. The ROS production is then involved in inducing the activation of the pro-inflammatory proteins IL-1β, IL-6, and TNF [90]. In addition, mitochondria-derived ROS play a crucial role in the activation of the inflammasome [91]. The inflammasome is a cytosolic multi-protein complex that regulates the activation of inflammatory caspases (caspase-1 and caspase-12) (Figure 2). Three different types of inflammasomes have been described: NALP1, NALP3, and IPAF. Of these, the NALP3 inflammasome is REDOX sensitive [92,93]. The activity of the NALP3 inflammasome is regulated by the thioredoxin binding protein (TXNIP, VDUP-1). TXNIP is bound to thioredoxin under non-inflammatory conditions. In the presence of ROS, it is released and can bind to NALP3 and activate the inflammasome. Activation of Caspase-1 and Caspase-12 induces cleavage and activation of IL-1β and IL-18 [94].

## 4. Gut Microbiome and the REDOX Status

The only site in vertebrates where a continuous activation of the immune system occurs is the gut. In humans, the intestine represents the largest contact surface of the body with the environment. The intestinal mucosa has two opposite functions: on one hand, nutrients have to be absorbed, and on the other hand, the infiltration of digestive enzymes, pathogens, and also commensal bacteria into the mucosa and circulation has to be prevented. Thus, the epithelial barrier with its diverse components (mucus, tight monolayer of epithelial cells, and the intestinal immune system) plays an essential role in homeostatic mechanisms. Although the intestine is the habitat for billions of bacteria, it is noteworthy that only a few individuals develop inflammatory diseases because of these commensal bacteria. Thus, in the physiologic situation, some mechanisms effectively differentiate between resident bacteria and invading pathogens and adjust their reaction accordingly. In contrast to other lymphoid organs, the intestinal immune system is not exclusively activated in the case of an infection. Moreover, a continuous immune response takes place. In a healthy organism, there is homeostasis, i.e., a balance of pro- and anti-inflammatory mechanisms. Only in the case of an actual infection, a temporary inflammatory reaction is initiated [8]. However, chronic intestinal inflammatory processes, such as those associated with chronic inflammatory bowel diseases (IBD), develop due to a complex disturbance of the immunological homeostasis.

Since the terms “microbiome” and “microbiota” have frequently been used as synonyms, they were clearly defined in 2020: The microbiota denotes the collection of microbial organisms within a community in an animal host and refers to the taxonomy of the microorganisms. The microbiome is the collection of microorganisms and their genes living in a particular environment. Thus, the microbiome contains the microbiota, its “activity”, and the surrounding environmental conditions [95], including bacteria, viruses, archaea, and fungi. The human gut microbiota is involved in various protective, structural, and metabolic functions and plays a central role in gut homeostasis and health. Here we focus on the role of bacteria in REDOX regulation in the gastrointestinal tract.

The gastrointestinal tract is a habitat for more than 100 trillion microorganisms with at least 1000 different species of bacteria [96]. Only about one-third of the gut microbiota constitutes a “common core”, whereas two-thirds of gut bacteria differ between individuals [97] and thus represent a kind of intestinal fingerprint. While being sterile at birth, the gastrointestinal tract is colonized thereafter, and a stable microbiota develops during the first two years of life. However, changes may occur over the course of a lifetime due to external factors such as health, age, and lifestyle [98]. Bacteroidetes (~15–50%), firmicutes (~20–50%), actinobacteria (<5%), and proteobacteria (<10%) constitute the predominant phyla of commensal bacteria in the human gut [99].

In addition to this plethora of microorganisms, the luminal side of the gastrointestinal tract is exposed to bacterial metabolites as well as to dietary components. This amount of antigen is countered on the intestinal side by the epithelial cells on one hand and the immune cells in the lamina propria on the other. It has long been known that the gut microbiome and its related low-molecular-weight metabolites play an essential role in the maturation of the host immune system but also in the homeostatic processes [100]. As a consequence, serious changes in the gut microbiome are closely associated with the development of inflammatory diseases such as IBD [101,102]. Of note, the gut microbiome cannot only induce local inflammatory reactions but moreover can induce systemic inflammation and intervene in the regulation of the function of extra-intestinal organs such as the brain, liver, and skin by inducing the production of specific metabolites [103,104,105,106,107,108]. Conversely, non-intestinal diseases can influence the interaction of gut bacteria with the epithelium, e.g., via the gut–liver axis in patients with liver cirrhosis [109]. This mutual interaction between the liver and the gut is mediated by the portal vein, which enables the transport of products originating from the intestine to the liver and the transport of bile and metabolites from the liver to the gut via the bile ducts. The mucosal and epithelial barrier of the intestine is the functional structure that serves as a connection point for the interactions between the intestine and the liver, which also limits the systemic spread of microbes. Control of microbial communities is critical for maintaining homeostasis of the gut–liver axis. As a result of this bidirectional communication, the liver has an impact on gut microbial communities [110]. However, emerging evidence has shown that the interaction of gut bacteria with intestinal epithelial cells or immune cells also exerts protective effects by regulating the REDOX status and thus contributes to the homeostasis.

### 4.1. Gut Bacteria and Chronic Inflammation

IBD are chronic diseases of the gastrointestinal (GI) tract that impair the quality of life. Crohn’s disease (CD) and ulcerative colitis (UC) are the main forms of IBD. Although it is widely known that IBD is characterized by an inappropriate immune response to environmental changes and by alterations in the intestinal microbiota, the underlying mechanisms of inflammation remain elusive [111]. The recognition of a REDOX imbalance in colonic tissues due to ROS overproduction has linked these reactive molecules to the development and the progression of IBD [34].

Intestinal epithelial and immune cells use pattern recognition receptors (PPRs) such as Toll-like receptors (TLRs) and NOD (nucleotide-binding oligomerization domain-containing protein) proteins to detect bacteria and their metabolites. These receptors, therefore, provide constant communication between the microbiota and the host [34]. Recognition of bacteria by host cells can lead to the release of O_2_·^−^ by NADPHoxidases and dual oxidase 2 (DUOX2). Increased expressions of NADPHoxidases, including NADPHoxidase 1, NADPHoxidase 2, and DUOX2, are therefore considered genetic risk factors for IBD [112]. O_2_·^−^ derived from the NADPHoxidases is rapidly converted to H_2_O_2_ [34] and re-enters the intestinal epithelial cells. This leads to alterations in signal transduction [113] as well as the induction of inflammatory processes [114]. Altered cellular signaling induces the expression of inflammatory cytokines, which in turn further increase NADPH-oxidase-dependent ROS production. In addition, the inducible nitric oxide synthase (iNOS) is induced [115,116]. The generation of O_2_·^−^ by NADPHoxidase and NO by NOS results in the generation of highly reactive and detrimental ONOO^−^. ONOO^−^ efficiently destroys bacteria but also oxidizes the plasma membrane of host cells, leading to the release of damage-associated molecular patterns (DAMPs) that amplify the inflammatory response [117]. Other immune cells such as leukocytes and monocytes are subsequently activated and further increase ROS accumulation [118]. This oxidative stress results in a chain reaction, which culminates in increased epithelial permeability [115,116]. It is noteworthy that oxidative stress can also be amplified directly by bacteria-produced ROS. For example, O_2_·^−^ is produced by bacteria such as *E. coli* [119,120]. In addition, bacteria, including *Lactobacillus* and *Bifidobacterium* [121,122], have been shown to produce H_2_O_2_ as a by-product of enzymatic reactions. In turn, bacterially produced H_2_O_2_ can increase inflammatory reactions and increase the permeability of the epithelium in IBD [123,124]. This increased permeability facilitates bacterial penetration into the lamina propria and contributes to sustained immune activation and ROS release, leading to an even more proinflammatory microenvironment and thus ROS-dependent perpetuation of the vicious cycle of chronic inflammation. In addition, the persistence of severe inflammatory processes in IBD has been shown to increase the risk of developing colitis-associated cancer [125]. This type of cancer differs from sporadic colorectal cancer in that it is chronic inflammation and an increased turnover of epithelial cells that drive tumor development. ROS produced during these inflammatory processes essentially contribute to the generation of dysplastic lesions [126,127]. Thus, it can be assumed that a regulation of the REDOX status in the gut has second-line effects on the development and progression of cancer.

### 4.2. Direct Effects of the Microbiome on the REDOX Status in the Gut

The human gut microbiota restricts the proliferation of pathogenic bacteria in the GI tract, activates the immune system, regulates nutrient utilization and host metabolism, and controls vitamin and enzyme production. The microbiota also produces short-chain fatty acids (SCFAs), ethanol, lactate, phenols, and succinate; degrades proteins and carbohydrates; and transforms bile acids [128,129].

**Figure 3:**
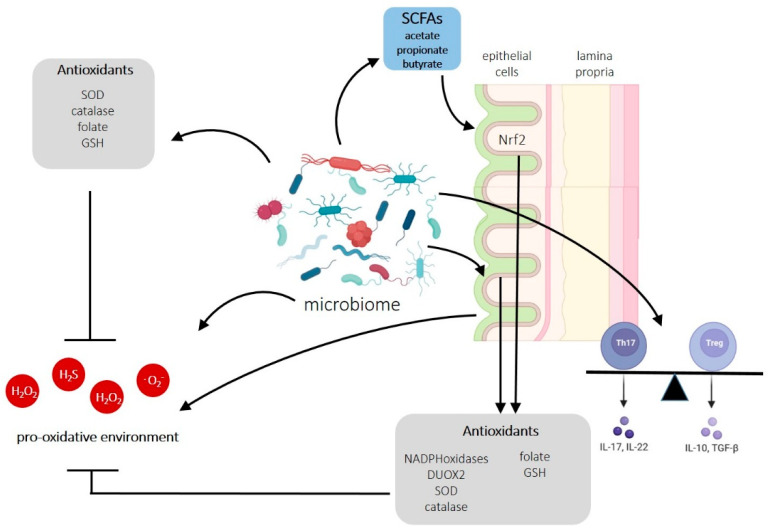
Direct and indirect influence of the gut microbiome on the intestinal REDOX status under physiological conditions. Gut bacteria can produce oxidative compounds but also have developed specific mechanisms to resist an oxidative environment. Moreover, gut bacteria can induce ROS production by intestinal epithelial cells. Beyond that, bacteria, especially probiotics, induce antioxidative activities in epithelial cells. This can occur directly or indirectly via SCFA production and Nrf2 activity. Furthermore, probiotics have beneficial effects on rebalancing immune responses mediated by T_h_17 and T_reg_ cells. The figure was created with BioRender.com (accessed on 1 May 2023).

Bacteria capable of producing ROS have developed specific mechanisms to resist an oxidative environment [130]. The ability to produce ROS, but also to counteract ROS, together with a variety of mechanisms to influence the REDOX status of the surrounding environment, makes bacteria key players in coordinating the intestinal REDOX equilibrium (Figure 3).

Generally, both commensals and pathogens can alter the production of ROS by host cells by modulating their mitochondrial activity or activation of NADPHoxidases [131]. In addition, during the degradation of sulfur-containing amino acids, pathogenic bacteria such as *Salmonella* and *Escherichia coli* can produce H_2_S, which impairs essential metabolic functions of colonic epithelial cells [105]. Increased levels of H_2_S can lead to a blockage of complex IV of the electron transport chain, resulting in epithelial damage, prevention of SCFA metabolism, and a disruption of the mucus barrier [33].

**Figure 4:**
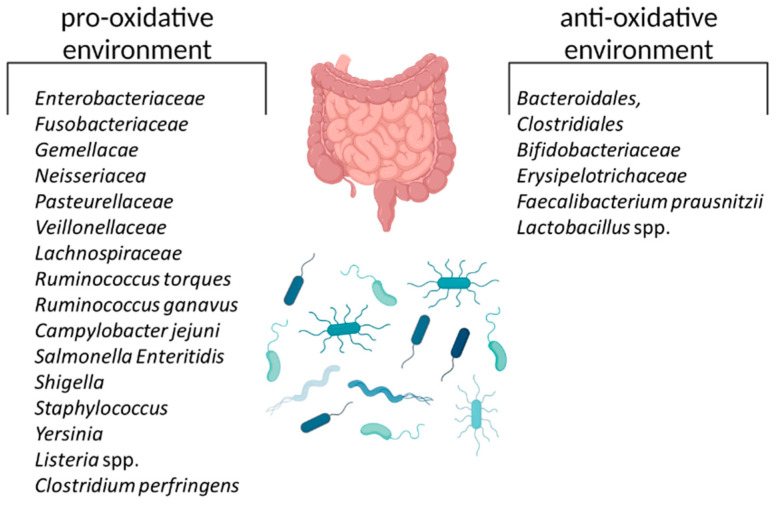
Overview of bacteria that favor a pro-oxidative state and bacteria that favor an anti-oxidative environment. The figure was created with BioRender.com (accessed on 1 May 2023).

Importantly, commensal bacterial communities can also essentially contribute to REDOX homeostasis in the gut [37]. A brief overview is given in Figure 4.

It is not surprising that these include predominantly probiotics, which are defined as live nonpathogenic microorganisms that, when administered in adequate amounts, are beneficial for the health of the host [132,133,134]. Probiotics include strains of lactic acid bacilli, nonpathogenic strains of *Escherichia coli* such as *Escherichia coli* Nissle 1917, and Saccharomyces boulardii [135], among others. Probiotics are known for several beneficial effects on the gut in treating gastrointestinal disorders such as ulcerative colitis [136], pouchitis [137], irritable bowel syndrome, and *Clostridioides difficile* infections [138,139]. Even so, there is currently no agreement between the guidelines of the various international professional societies on the use of probiotics in the treatment of gastrointestinal diseases [136,140].

Of note, probiotics are also able to counteract high ROS levels by inducing anti-oxidative processes via several mechanisms [132]: Probiotics produce their own anti-oxidases such as SOD or catalase, and they also generate anti-oxidative metabolites such as folate and GSH. Moreover, probiotic lactic acid bacteria produce antioxidant metabolites such as exopolysaccharides, carotenoids, ferulic acid, or histamine, which reduce oxidized molecules and thereby contribute to the REDOX equilibrium [141]. Furthermore, probiotics affect host cells by inducing their anti-oxidative capacities (activation of host SOD and catalase and upregulation of the production of folate and GSH) and, simultaneously, dampening the activities of ROS-producing enzymes [37,132,141]. The latter actions are based on the regulation of several signaling pathways in the host cell, including Nrf2 (nuclear erythroid 2-related factor 2), SIRT, MAPK, and PKC [141].

### 4.3. Indirect Effects of the Microbiome on the Redox Status in the Gut

Besides direct intervention in the production of ROS and antioxidants, bacteria use a range of options to indirectly control the intestinal REDOX state. Commensal bacteria are producers of a set of metabolites, which can influence the REDOX status in the intestine: formyl-peptides, reactive nitrogen species (RNS), and SCFAs.

ROS are generally considered to be destructive pro-inflammatory molecules. However, they also trigger a number of important physiological functions, such as cell proliferation and migration. In addition, ROS also play an important role in angiogenesis [35].

Here it is crucial where and how much ROS is produced. NADPHoxidase 1 plays a key role in this context. NADPHoxidase 1 is expressed in epithelial cells and produces many times fewer ROS than NADPHoxidase 2 from immune cells. Some strains of *Lactobacilli* induce NADPHoxidase 1. This leads to reversible oxidation and inactivation of the tyrosine phosphatases LMW-PTPase and SHP-2 resulting in the activation of the focal adhesion kinase (FAK). This promotes focal adhesion and accelerates wound healing in the intestine [31,142,143]. Furthermore, the influence of ROS on proliferation and epithelial growth can be detected. It has been shown that ROS induced by commensal bacteria also lead to the inactivation of ERK phosphatase DUSP6, resulting in enhanced ERK phosphorylation and activation of the ERK signaling pathway [144,145]. Thus, it can be stated that bacterial induction of NADPHoxidase 1 plays an important role in the growth and differentiation of intestinal epithelial cells [31].

Commensal-derived formylated peptides bind to G protein receptors on immune cells such as macrophages and neutrophils, as well as epithelial cells leading to inflammatory processes and an enhanced ROS generation in the gut via activation of NADPHoxidases [105,146]. Moreover, *Lactobacilli* and *Bifidobacteria* as well as *Streptococcus* and *Bacilli* are capable of synthesizing NO [147].

**Figure 5:**
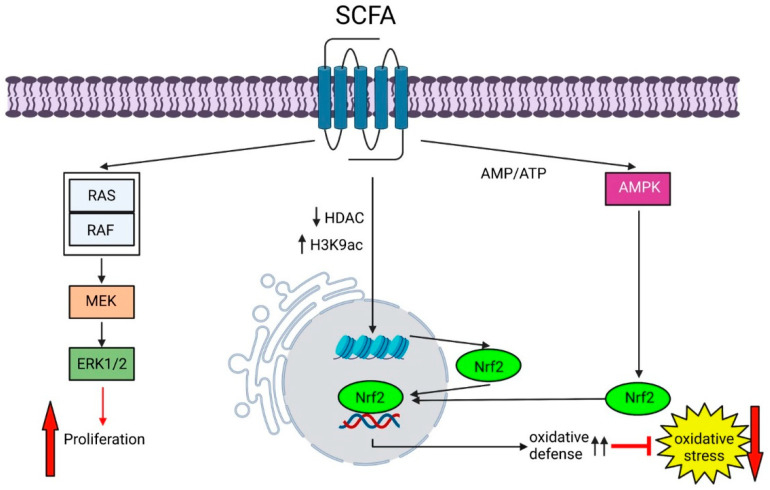
SCFAs induce proliferation and strengthen oxidative defense. SFCAs bind to FFARs and activate the RAS/RAF/MEK/ERK signaling pathway, inducing proliferation. Furthermore, stimulation of the FFAR receptor by SCFA butyrate shifts the AMP/ATP ratio and induces AMPK. Activated AMPK induces translocation of Nrf2 into the nucleus. In addition, inhibition of HDACs also increases Nrf2 synthesis. Thus, Nrf2-mediated activation of oxidative defense mechanisms occurs. SCFA: short- chain fatty acid; FFAR: free fatty acid receptor; RAS: rat sarcoma virus protein; RAF: rapidly accelerated fibrosarcoma protein; MEK: mitogen activated kinase; ERK: extracellular signal-regulated kinase; AMPK: AMP-activated protein kinase; HDAC: histone deacetylase; Nrf2: Nuclear Factor Erythroid 2-related Factor 2. The figure was created with BioRender.com (accessed on 1 May 2023).

Anaerobic fermentation of dietary fibers by bacteria produces the SCFAs acetate, propionate, and butyrate [102,105,148]. These metabolites have a variety of functions, ranging from energy supply for epithelial cells to effectors on neuronal development and other physiologic functions of organs via systemic circulation [105]. SCFAs have been shown to enhance tight junction proteins between endothelial cells [149], and intestinal epithelial cells [150,151,152]. Thus, they exert a direct protective effect on both the stabilization and the recovery of the intestinal epithelial barrier. Most importantly, SCFAs have been shown to modulate oxidative stress. Specifically, SCFAs can activate the antioxidant defense system via the Keap1-Nrf2 defense pathway [149,153,154]. Nrf2 is a key transcription factor of the cellular antioxidant defense by controlling more than 200 genes (Figure 5) [155]. By enhancing antioxidant defenses, SCFAs can reduce the mitochondrial damage caused by ROS and improve mitochondrial function. This protects the mitochondrial metabolism and enables a better energy supply (ATP) to the cells. In addition, the respiratory chain of the mitochondria is also protected from oxidation. Damage to the respiratory chain leads to an electron release, which leads to further ROS generation and starts a chain reaction of increased mitochondrial damage and ROS generation. Thus, SCFAs form a protective shield that defends against oxidative and mitochondrial stress.

Another non-negligible indirect effect of the microbiome on the REDOX status in the gut is the regulation of immune homeostasis. Commensal bacteria are essential regulators of intestinal immune homeostasis by maintaining the balance of pro- versus anti-inflammatory cytokine production by T_h_17 and T_reg_ cells [102,156]. The probiotic *Lactobacillus acidophililus* has been shown to restore a homeostatic ratio of both T_h_17 and T_reg_ cells and pro- and anti-inflammatory cytokines in a mouse model of colitis [157]. Concordantly, *Clostridia* and *Bacteroides* augmented anti-inflammatory responses by the induction of T_reg_ cells [158,159,160]. Since an excess of pro-inflammatory cytokines leads to increased ROS production as part of the inflammatory response [161], restoring the balance between T_h_17 and T_reg_ cells by specific bacteria builds a second line of defense against excess ROS production in the epithelium.

Vice versa, NADPHoxidases in the intestinal epithelium have an impact on the gut microbiome. A partial or complete inactivation of NADPHoxidases in the epithelium results in an altered H_2_O_2_ gradient in the mucus layer and, consequently, a change in the composition of the microbiome [34]. Interestingly, the inactivation of Nox1–4 together or individually increased the abundance of facultative aerobic bacteria, especially Firmicutes [162]. This example impressively demonstrates how closely epithelial REDOX mechanisms and the intestinal microbiome are interlinked, so that insufficient ROS production can be compensated by the expansion of H_2_O_2_-producing bacteria [34].

### 4.4. Therapeutic Interventions Reconstituting a Physiologic REDOX State in the Gut

Given the major influence of the microbiome on the intestinal REDOX status, it is reasonable to consider how the ROS balance can be specifically supported by bacteria. Thus, enhancing antioxidant-producing bacteria will shift the ROS ratio. Restoring the REDOX equilibrium in the gastrointestinal tract, therefore, involves treatment strategies to enhance the microbiome exerting direct or indirect antioxidative effects as depicted above. Thus, excessive ROS production as it occurs in several gastrointestinal diseases such as IBD, intestinal infections, ischemic damage, and colorectal cancer [37] might be attenuated by the administration of specific antioxidant-producing bacteria. Again, it is the group of probiotics that appear of special interest in this context.

Firmicutes such as *Lactobacillus*, *Bifidobacterium*, and *Butyricicoccus* are known to exert protective and anti-inflammatory effects on the intestinal epithelium [34]. Bacteria of the genus *Butyricicoccus* are reduced in the stool of patients with inflammatory bowel disease, and administration of *Butyricicoccus pullicaecorum* reduced mucosal lesions and inflammation in a rat model of colitis [163].

In addition to the long-known protective probiotics *Lactobacillus* and *Bifidobacterium*, “novel” probiotics are discussed, including anaerobic SCFA-producing strains [37]. As outlined above, SCFAs play a crucial role in activating the antioxidant defense system and exert several additional protective functions to maintain intestinal homeostasis. Therefore, an increase in SCFA-producing bacteria not only enhances antioxidant mechanisms but also contributes to the stabilization of the epithelial barrier and thus to reduced inflammatory responses. One candidate might be *Faecalibacterium prausnitzii*, which is reduced in samples from patients with gastrointestinal diseases and metabolic disorders such as IBD, irritable bowel syndrome, colorectal cancer, obesity, and celiac disease [164]. In a recent study, *Faecalibacterium prausnitzii* not only enhanced SCFA production but also had ameliorating effects on non-alcoholic fatty liver disease in mice [165]. Another option to support the growth of SCFA-producing bacteria is to supplement the diet with prebiotics. Prebiotics are defined as non-digestible fibers and other dietary compounds such as glycans or non-digestible carbohydrates that are selectively utilized by beneficial microorganisms in the gut. Thus, providing specifically tailored dietary compounds will create a growth advantage for SCFA-producing microbiota and enhance antioxidative processes without the need to culture beneficial anaerobic intestinal bacteria in vitro.

Last but not least, every anti-inflammatory effect of beneficial bacteria will have a second-line effect on ROS production in the gut. Concordantly, enrichment with probiotics will (i) shift the composition of the microbiome, (ii) reduce pro-inflammatory reactions by enhancing T_reg_ responses, and (iii) reduce ROS levels due to diminished inflammation. *Faecalibacterium prausnitzii*, which is reduced in patients with IBD [166], revealed anti-inflammatory effects in Crohn’s disease [102,167]. Moreover, microbial products such as polysaccharides from *Bacteroides fragilis* as well as protein compounds from *Faecalibacterium prausnitzii* have been shown to induce IL-10-producing T_reg_ in mice [102,168].

Taken together, both a dysbiosis of the gut microbiome and a REDOX imbalance are involved in the pathogenesis of a range of diseases, i.e., IBD [34,37], non-alcoholic fatty liver disease (NAFLD) [169], neurodegenerative disorders [103], inflammatory skin disorders [170], and cancer [171]. Therefore, regulating gut immunity by optimizing its microbiome and REDOX status appears to be quite an attractive novel option to treat intestinal or gut-associated diseases.

## 5. Conclusions

ROS production in the gut is a double-edged sword: on one hand, it is an indispensable mechanism in the defense against pathogens and mucosal healing. On the other hand, excessive ROS production can have detrimental effects on mucosal integrity and epithelial barrier function.

The effects of ROS production are a matter of dose, which is also true for pro- and anti-inflammatory mechanisms via cytokines, T-cell populations, or responses to bacterial components in the gut [172,173]. Finely adjusted ROS production has vital functions as it helps to defend against pathogens and induce repair mechanisms. Exaggerated ROS levels in the context of chronic inflammation can cause severe tissue damage if they are not counteracted by anti-oxidative mechanisms.

Oxidative stress due to exaggerated ROS production is a vital process in the body, but chronic exposure can lead to the oxidation of biomolecules and activation of inflammatory signaling pathways, resulting in genomic instability and the dysregulation of gene and protein expression and tumor initiation or cancer cell survival [127,174]. In addition, chronic intestinal diseases such as IBD increase the risk of tumor development by the promotion of cell proliferation and angiogenesis [127,175]. A recent study indicated that a “healthy” gut microbiome contains bacteria suppressing tumor growth by antioxidative metabolites [176]. Thus, an ROS-balanced microbiome can also contribute to tumor suppression.

The constant interaction of the host with its microbiome ensures the maintenance of the highly complex ecosystem in the gastrointestinal tract. This principle of host–microbiome interaction includes the regulation of the REDOX equilibrium. To take advantage of this interaction, oxidative conditions in certain intestinal diseases can be counteracted by the enrichment of bacteria with enhanced anti-oxidant activity. Thus, the modulation of the individual microbiome will be an important issue regarding future therapy for gastrointestinal disorders, cancer, and other diseases.

## Data Availability

Not applicable.
